# Fusing Geometric and Semantic Features via Cosine Similarity Cross-Attention for Remote Sensing Scene Classification

**DOI:** 10.3390/s26051613

**Published:** 2026-03-04

**Authors:** Xuefei Xu, Chengjun Xu

**Affiliations:** 1 School of Information Engineering, Shanghai Dianji University, Shanghai 201306, China; xuxf@sdju.edu.cn; 2 School of Artificial Intelligence, Jiangxi Normal University, Nanchang 330022, China; 3 School of Remote Sensing and Information Engineering, Wuhan University, Wuhan 430072, China

**Keywords:** cross-level attention, dual-branch network, Lie Group, multi-scale fusion, remote sensing scene classification

## Abstract

**Highlights:**

**What are the main findings?**

- The extraction and integration of multi-level features—such as shallow and high-level features—significantly enhance the accuracy of remote sensing scene classification.
- A bidirectional cross-attention mechanism effectively fuses shallow and high-level features while suppressing redundant information and improving feature discriminability.

**What are the implications of the main findings?**

- Shallow and high-level features capture complementary information: shallow features preserve physical structures and local details (e.g., edges and textures), while high-level features encode rich semantic content.
- The fusion of these heterogeneous features enhances the overall representational capacity of the model, leading to more robust and interpretable scene classification, especially in complex environments with high intra-class variation and inter-class similarity.

**Abstract:**

High-resolution remote sensing image scene classification (HRRSI-SC) is crucial for obtaining accurate Earth surface information. However, the task remains challenging due to significant background interference, high intra-class variation, and subtle inter-class similarities. Convolutional neural networks (CNNs) are constrained by their local receptive fields, which limits their ability to capture long-range spatial dependencies. On the other hand, Vision Transformers (e.g., ViT-B-16) excel at global feature extraction but often suffer from high computational complexity and may lack the inherent inductive biases for local feature modeling that CNNs possess. To address these limitations, this paper proposes a cross-level feature complementary classification framework based on Lie Group manifold space, termed CBCAM-LGM. Within the proposed CBCAM-LGM framework, multi-granularity features are first distilled via a global average pooling layer to suppress redundant information. The core of our approach, the cross-level bidirectional complementary attention module (CBCAM), then enables the adaptive fusion of features from both branches through a cross-query attention mechanism. Furthermore, by employing parallel dilated convolutions and a parameter-sharing strategy, the model captures multi-scale contextual information by sharing a single set of convolutional weights, which reduces the computational complexity to merely 1.21 GMACs while preserving multi-scale representation with minimal parameter overhead. Extensive experiments on challenging benchmarks demonstrate the model’s efficacy, as it achieves a state-of-the-art classification accuracy of 97.81% on the AID, surpassing the ViT-B-16 baseline by 1.63%, while containing only 11.237 million parameters (an 87% reduction). These results collectively affirm that our model presents an efficient solution characterized by high accuracy and low complexity.

## 1. Introduction

The advancement of remote sensing technology enables the acquisition of high-resolution remote sensing images (HRRSI) [1,2], providing multimodal data crucial for disaster prediction [3,4,5]. As a core task in interpreting HRRSI, remote sensing scene classification (RSSC) necessitates the extraction of distinctive features from complex scenes. Key persistent challenges encompass: (a) ambiguous class distinctions characterized by high inter-class similarity and intra-class variation [6], (b) the manifestation of objects across multiple scales [7], and (c) irregular spatial layouts [2]. Addressing these complexities requires innovative hierarchical representation learning frameworks to enhance RSSC robustness.

To address these challenges, present RSSC models can be broadly categorized into the following three primary groups [8,9]:

Such models rely primarily on handcrafted features, such as Scale-Invariant Feature Transform (SIFT), Local Binary Patterns (LBP), and Histogram of Oriented Gradients (HOG), or coded extensions [8]. Empirical validations include SIFT [10] for building change detection and LBP for cloud classification [11]. Extended implementations integrate CNN-encoded binary patterns [12] and color histogram quantization [13]. Although effective for rigid transformation robustness and low in computational cost, their semantic abstraction capacity remains constrained, failing to model complex nonlinear patterns like agricultural textures or degraded urban features.

To advance beyond shallow representations, mid-level feature models employ coding schemes for localized semantic enrichment. Core methodologies comprise: (1) fundamental encoders, such as Bag-of-Visual-Words (BoVW) scene classifier [14]; (2) distribution-based encoders, such as Fisher vectors [15,16]; and (3) spatial context models, such as Spatial Pyramid Matching [17]. Subsequent refinements incorporate the Vector of Locally Aggregated Descriptors [18] and Locally Constrained Linear Coding encoding [19]. While outperforming shallow features in semantic preservation, they fundamentally fail to adaptively model complex geometries (e.g., irregular croplands, forests) and suffer from rigid dimensional constraints, critically limiting cross-scale generalization [20,21].

High-level feature-based models such as CNN [22] and Transformer [23] can extract high-level abstract features with semantic information from raw pixels by end-to-end deep learning, compared to shallow and mid-level models in understanding complex scenes. CNN models like ResNet [24] and MobileNets [25] extract local features and spatial hierarchical modeling, while Transformers like ViT [23], Swin-Transformer [26], and SCViT [27] capture global context by self-attention. Hybrid models combining CNN and Transformer strengths have promising possibilities for improved classification performance. Xu et al. [28] presented a joint Lie Group-CNN model that maintains geometric invariance in Lie Group space with an accuracy of 93.6% on the UCMerced dataset. STConvTeXt [29] incorporates weighted normalized CNN preprocessing with a cross-space interaction module, which is 2.3 times faster than Swin-Transformer. LWGANet [30] constructs a lightweight group attention skeleton and achieves a classification accuracy of 91.4% with 5.7 M parameters. These models improve the ability to interpret complex scenes, transitioning from individual local or global modeling to collaborative optimization [31]. Nevertheless, while deep networks demonstrate remarkable capability in learning rich feature representations from data, we posit that explicitly incorporating strong inductive biases derived from domain knowledge (such as Lie Group structures) can serve as a valuable complement. Such integration is hypothesized to provide more stable and interpretable low-level cues, potentially enhancing both learning efficiency and final performance. Recently, parameter-efficient foundation models such as SpectralX [32] have shown promising domain generalization capabilities for spectral remote sensing, highlighting the trend towards efficient and adaptable architectures.

As the research progresses and the remote sensing scenes become more complex, high-level feature models face pressing problems:

Lack of feature extraction: Many current approaches use deep learning to extract semantic features of a scene, ignoring shallow features, leading to a lack of feature extraction, neglecting location, color, texture, edge features, etc. [33].

Traditional CNNs like ResNet or MobileNets, with their fixed-size kernels and limited receptive fields, struggle to model large spatial dependencies, leading to a lack of global semantics. Dilation, convolution, and attention [34] have been proposed to improve the local receptive field, but merging features with these mechanisms usually involves basic concatenation or weighted sum.

The tradeoff between efficiency and generalization power is difficult for Transformer models ViT, Swin-Transformer, and SCViT. These models focus on self-attention to capture global context but quadruple computational complexity with image resolution. ViT-B-16 [23] on a 512 × 512 image can have up to 3.25 GMACs and lack local inductive bias in CNNs. ViTAE [35] and V2T-ViT [36] introduce intrinsic biases (e.g., hierarchical token aggregation and conv–attention hybrids) to mitigate Vision Transformers’ data inefficiency. However, both still rely on large-scale pretraining to avoid overfitting, limiting their applicability in data-scarce remote sensing scenarios [37].

In summary, the core motivation of our work stems from a key observation: in remote sensing scene classification, shallow geometric features (e.g., edges and textures) and deep semantic features (e.g., objects and scene semantics) are inherently complementary, yet existing methods fail to integrate them in an efficient and adaptive manner. CNNs excel at extracting local details but suffer from limited receptive fields; ViTs capture global dependencies but incur high computational costs and lack explicit modeling of local geometric structures, while simple concatenation or weighted summation cannot effectively handle complex correlations and redundancies between heterogeneous features. To this end, we propose the Cross-Level Feature Complementary Classification (CBCAM-LGM) framework, whose central idea is to achieve dynamic and adaptive fusion of shallow geometric and deep semantic features within a heterogeneous dual-branch architecture via a cosine similarity-based bidirectional cross-attention mechanism. This design allows us to simultaneously leverage the geometric invariance prior brought by Lie Group space and the powerful semantic extraction capability of deep learning while effectively suppressing redundant information and enhancing feature discriminability without significantly increasing computational overhead.

The main contributions of the framework are:

1. Heterogeneous two-branch feature extraction architecture:

The dual-branch architecture proposed in this paper is inherently heterogeneous. This represents a fundamental departure from traditional homogeneous multi-scale architecture. The “heterogeneity” in our work is embodied in its two distinct branches: one relies on prior knowledge-driven, fixed feature engineering (Lie Group structure), while the other is based on data-driven, learnable feature discovery (deep convolutional networks). Our objective is to fuse these two disparate cognitive paradigms, rather than simply combining variants of the same type of operation. The Lie Group covariance matrix encodes rotationally invariant texture features like edges and color distributions for shallow geometric branching, position, color, Canny edges, and Gray-Level Co-occurrence Matrix (GLCM) texture features. To capture multi-granularity semantics, we developed a multi-branch model where each branch replaces common convolution blocks with parallel dilated convolution blocks using multi-level convolution kernels 3 × 3, 5 × 5, and 7 × 7. Large convolution kernels expand local receptive fields. Expanding local details with global dependencies changes the dilation rate as depth increases. Cross-layer residual connectivity mitigates information loss in deep features by bridging shallow and deep representations through skip connections.

2. Cosine similarity-based bidirectional cross-query attention mechanism (CBCAM):

To address global long-range dependencies between dual-branch feature maps, we propose a Bi-directional Cross-Query Attention (BCQA) mechanism. BCQA merges cross-branch contextual information via cosine similarity-weighted feature aggregation while reducing dependency redundancy. The key implementations include unifying spatial-channel dimensions (H × W × C) through tensor combination, enhancing image block embedding efficiency, generating attention maps via normalized cosine similarity, and enabling adaptive cross-layer fusion with residual gating.

3. Lightweight and efficient design:

The design of parallel dilated convolution with cross-stage residual connection comprises three key components: parallel dilated convolution at multiple scales, which expands contextual information; grouped feature interaction for efficient information exchange; and BCQA feature fusion, facilitating information flow. Parallel dilated convolution (PDConv) effectively expands the receptive field and captures contextual feature information by introducing gaps in the convolution kernel. Grouped features interaction allows the network to gather and integrate information from different sources or granularities, promoting the complementarity and interaction between local and global information. BCQA feature fusion information flow further enhances the network’s capability by effectively fusing and propagating the extracted features. Achieving high accuracy at 1.21 GMACs computational cost with 40% inference acceleration on an NVIDIA RTX 4090 GPU compared to ResNet50.

## 2. Related Work

1. Lie Group-based feature learning methods

Lie Groups and Lie Algebra [38] gives the theory of differential manifold structure of feature space and mathematical tools for geometric invariant modeling. Xu et al. [28] developed a joint Lie Group-convolutional model. This model utilizes a feature representation based on Lie Group covariance matrices to improve rotational and scale invariance through manifold alignment. They optimized the convolutional kernel parameters, achieving 98.67% accuracy on the UCMerced dataset. They then developed a supervised adversarial Lie Group feature learning network [39] to generate data samples of different scales, which achieves better scene classification accuracy with limited data samples. Zhang et al. [40] proposed a Lie Group-based model that integrated multi-scale features and hybrid attention. Combining shallow sense features with high-level semantic features through a feature fusion model enables a more comprehensive scene representation, and the model achieved 97.29% overall accuracy on the AID. Even with these advances, current methods do not exploit complementary information of cross-level features and face problems such as redundant feature interference in complex cases.

2. Multi-scale feature fusion methods

The evolution of multi-scale feature fusion techniques has significantly contributed to the stability of RSSC. Initial work by Xu et al. [9] demonstrated the efficacy of feature-level fusion for dimensionality reduction and representation enhancement, achieving 97.72% accuracy on AID. This direction was dependent on [41] through the integration of socio-economic semantic features and attention-based pooling, with adaptive fusion effectively handling complex scenarios characterized by high interclass similarity and large intra-class differences. The paradigm further evolved with the global–local, three-branch model in [42], where a dedicated fusion module with dual attention mechanisms achieved a high accuracy of 97.31% with moderate parameters of 12.216 M. The latest advancement, presented in [43], leverages Lie Group manifold learning to formulate a contextual attention mechanism, marking a substantial 7.13% improvement on URSIS and pointing to a promising direction for feature fusion.

3. Optimization of attention mechanisms

The attention improves the response of critical regions by filtering features. Xu et al. [1] proposed a spatial attention mechanism that combines Lie Group theory with CNNs. This approach preserves multi-level features and suppresses irrelevant information. Li et al. [43] proposed a global and local attention mechanism merging local details with global context. Sitaula et al. [44] proposed an enhanced attention module merging local details with global context for classification stability. Zhao et al. [45] proposed the local context attention block model. This model enhances feature discriminability by merging local features with global context information, utilizing a mechanism that splits attention blocks. Wang et al. [46] proposed the MSRes-SplitNet model, merging local features and global information by splitting attention blocks, which reduces parameters while maintaining performance. While SENet [47] pioneered channel attention in CNNs by using global average pooling to weight channels, its reliance on this operation restricts spatial awareness. Woo et al. [48] proposed a convolutional block attention module, which combines channel and spatial attention to improve feature discrimination. However, this dual-attention mechanism incurs high computational complexity when processing high-resolution images. Recently, Lie Group theory and attention mechanisms have been explored, such as Zhao et al. [49], who proposed an enhanced attention model; Zhang et al. [40], who proposed a mixed attention mechanism; and Xu et al. [6], who proposed a contextual attention mechanism. These models gained attention for their robustness but still have high training complexity.

In recent years, cross-attention mechanisms have gained widespread adoption in various remote sensing vision tasks due to their exceptional performance in feature alignment and information fusion. For instance, Yao et al. [50] applied cross-attention within a dual-stream architecture for hyperspectral image super-resolution, enabling bidirectional information transfer between multimodal images. Wei et al. [51] employed a cross-modal attention mechanism in remote sensing image captioning to achieve fine-grained semantic alignment between visual regions and bilingual text. Ma et al. [52] introduced a cross-layer attention model for change captioning tasks, effectively capturing key change features in bi-temporal images. These advancements demonstrate the considerable potential of cross-attention in handling the multi-source, multimodal, and temporal characteristics of remote sensing data. Nevertheless, existing approaches predominantly utilize cross-attention for front-end and back-end information bridging in specific tasks, such as image-to-image or image-to-text cross-modal interactions. In the critical field of remote sensing scene classification, the application of cross-attention principles to optimize feature coordination among heterogeneous branches within a network, rather than merely achieving inter-modal alignment, remains an underexplored area.

4. Lightweight modeling

Lightweight models are important for edge computing in remote sensing applications. MobileNetV2 by Sandler et al. [53] reduces the number of parameters by refactoring the residual design (91.23% accuracy on AID). The convolutional neural networks meet Vision Transformers models by Guo et al. [54], which integrates the strength of CNN and Transformer with 21 M parameters. Zhang et al. [55] develop a lightweight discriminative model incorporating multi-dilated spatial pooling, with parameters reduced to 3.5M. Concurrently, Xu et al. [28] achieve 2.07 M parameters in their Lie Group-CNN joint representation through streaming space computation. LWGANet by Lu et al. [30] (13M parameters, 0.935 GMACs) and STConvNeXt [29] by Liu et al. achieve 96.25% accuracy on AID with 10.52M parameters. Beyond these, recent advances have introduced more complex architectural paradigms to enhance lightweight models. For instance, Capsule Attention Networks [56,57] have been explored to better model part-whole relationships in complex scenes. Similarly, clustering techniques like Doubly Stochastic Graph Projected Clustering [58,59] offer novel pathways for unsupervised feature learning in hyperspectral data, while Multi-order Dynamical Low-Rank Tensor Approximation [60] methods provide powerful tools for compressing and analyzing high-dimensional remote sensing data streams. Nevertheless, prevailing lightweight approaches often sacrifice classification capability. For instance, MobileNetV2 not only shows a significant accuracy drop (6.06%) [53] but also struggles with distinguishing semantically similar yet geometrically distinct scenes, due in part to limited multi-scale integration. A more critical bottleneck lies in constrained multi-scale feature interactions within lightweight architectures, which necessitate higher computational expenditures for performance compensation. CMT has achieved remarkable performance; its computational cost of 2.1 GMACs poses a limitation for deployment in resource-constrained environments [54].

Compared to the works discussed above, the distinctiveness of our proposed CBCAM-LGM framework is threefold. Firstly, it constructs a genuinely heterogeneous dual-branch architecture—one branch based on fixed Lie Group feature engineering and the other on learnable deep convolutional networks—as opposed to homogeneous multi-scale variants. Secondly, it introduces the cosine similarity-based bidirectional cross-query attention (CBCAM) as the core mechanism for fusing these heterogeneous features, which differs from existing self-attention or channel/spatial attention modules. Thirdly, through its parameter-shared Parallel Dilated Convolution (PDConv) design, the framework achieves effective multi-scale context capture with minimal computational overhead, offering a new perspective on lightweight and efficient model design.

## 3. Methodology

### 3.1. Overall Framework and Pipeline

**Figure 1:**
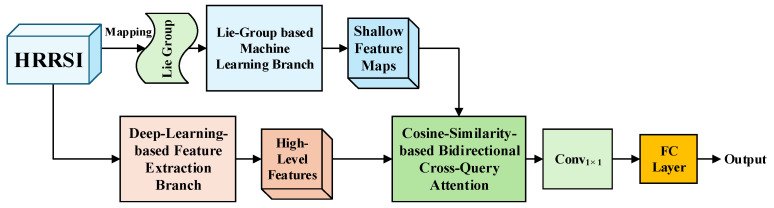
The two-branch collaborative learning framework.

Our proposed CBCAM-LGM aims to synergistically integrate geometrically invariant shallow features and semantically rich deep features for HRRSI scene classification. Figure 1 illustrates the overall architecture, which comprises two heterogeneous branches and a central fusion module. The end-to-end processing pipeline can be summarized as follows:

**Input:** A HRRSI $I\in R^{H\times W\times C}$.

**Shallow Feature Branch (Path A):** Extracts spatial coordinates, YCbCr color, Canny edges, and GLCM textures. Encodes them into a Lie Group covariance.

**Deep Feature Branch (Path B):** Extracts high-level, multi-scale semantic features. Uses a stack of parameter-shared Parallel Dilated Convolution (PDConv) blocks to capture contextual information at multiple receptive fields.

**Feature Alignment:** Aligns shallow and deep features that are spatially and channel-wise aligned via upsampling and 1 × 1 convolution.

**CBCAM Fusion Module:** Applies the cosine similarity-based bidirectional cross-attention mechanism to enable complementary information flow between the two branches.

**Output:** Fused features are pooled and fed into a classifier for scene prediction.

### 3.2. Shallow Feature Extraction Branching Based on Lie Group Machine Learning

The Lie Group-based shallow feature extraction described in this section builds upon the theoretical foundation of Lie Group covariance descriptors, primarily following the work of Xu et al. [28]. The novelty of our work lies in its integration as one heterogeneous branch within a dual-path framework and, more importantly, in the design of a novel interaction mechanism between this branch and the deep learning branch.

Shallow branch extraction, as shown in Equation (1), is optimized using physically interpretable binding properties based on previous studies [1,2,3]:

(1) $$F(x,y)={\left[x,y,Y,C_{b},C_{r},Canny(x,y),GLCM(x,y)\right]}^{T}$$

where T is the transformation matrix mapping features to a shared Lie Group manifold.

1. Preservation of identity:

Spatial coordinates (x, y) improve the sense of target location for location-sensitive tasks like the detection of roads or building clusters [4].

2. Optimization of features:

The separation of luminance (Y) and chrominance (Cb and Cr) components in the YCbCr color space reduces the impact of varying lighting conditions and enhances robustness in cloudy/shadowed scenes [1], as demonstrated in Equation (2).

(2) $$\left\{\begin{array}{c} Y=0.299R+0.587G+0.114B\\ C_{b}=-0.1687R-0.3313G+0.5B+128\\ C_{r}=0.5R-0.4817G-0.0813B+128 \end{array}\right.$$

We incorporate Canny edge detection [61] with adaptively determined thresholds by applying Otsu’s method, where the low threshold is set to 0.4 times the high threshold. Canny edge detection uses Otsu’s method applied per image to determine adaptive thresholds, ensuring robustness to illumination variations across scenes. We extract scale-invariant structural features to enhance multi-scale edge localization accuracy. This is particularly effective for geometrically sensitive categories (e.g., bridges and airport runways) [7], aligning with the scale-invariant keypoint principle.

GLCM texture features are second-order statistics representing texture spatial distribution rather than local descriptors such as LBP and are more suitable for forest/agricultural land classification. The GLCM texture feature computation draws on Avramovic et al.’s [62] modeling of the block-based semantic classification method. In our implementation, GLCMs are computed with a 1-pixel offset across four directions (0°, 45°, 90°, and 135°), and the final features (e.g., contrast and homogeneity) are averaged to ensure rotation invariance. We also experimented with larger offsets (3–5 pixels) to capture longer-range textural patterns. While these showed slight improvements in homogeneous categories like forest and farmland, the overall accuracy gain was marginal (<0.3%). Thus, we retained a 1-pixel offset for efficiency and fine-texture emphasis. This enhances the continuity of the representation of the spatial distribution of the texture through the localized block statistic properties. Table 1 lists the GLCM (x, y) components applied: correlation; contrast (vegetation density); angular second moment (farmland texture); entropy (natural disorder); and inverse difference moment (cloud regions).

Computation of the Lie Group regional covariance descriptor is summarized as follows. We first construct a 7-channel low-level feature tensor by stacking normalized spatial coordinates (x, y), YCbCr components (Y, Cb, and Cr), an edge magnitude map from Canny, and an aggregated GLCM texture channel (computed over multiple directions and averaged). For each local region, we form 7-D feature vectors and estimate a covariance matrix (with mean-centering and a small diagonal regularizer for numerical stability). Since the covariance matrix is symmetric positive definite, we apply a log-Euclidean mapping to obtain an element in a Euclidean space for subsequent learning and matching. Regional second-order statistics provide a compact way to model diverse textures, while multi-directional aggregation helps reduce sensitivity to rotation.

### 3.3. Deep Learning Based High-Level Feature Extraction Branching

The concept of Parallel Dilated Convolution (PDConv) is inspired by prior work on multi-scale context aggregation [34]. Our key modification, however, is the introduction of a ‘parameter-sharing strategy,’ where multiple convolutional layers with different dilation rates share the same set of convolutional kernel weights. This design significantly reduces the number of parameters and computational complexity without compromising multi-scale representational capacity.

As the depth of the web model increases, it can extract more detailed and abstract high-level semantic features. However, over-deep network models usually suffer from the following risks: (1) overfitting (model performs well on the training set but does not generalize in real tests); (2) model degradation (model performance decreases rather than improves with network layer increases); and (3) high computational burden—significant computational costs arise from complex network architectures. Their high parameter counts and feature dimensions directly impact efficiency, while hardware requirements surge accordingly. To address these limitations, we propose a lightweight deep learning architecture serving as the high-level feature extraction branch.

**Figure 2:**
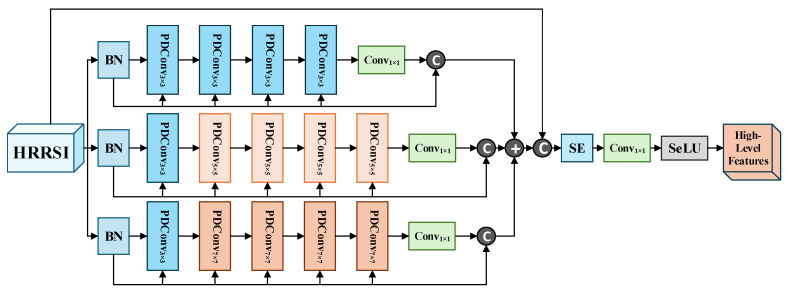
Deep learning-based high-level feature extraction architecture.

Figure 2 shows that this architecture addresses the overfitting of models through three PDConv branches. In the first branch, deep semantic features undergo 3 × 3 PDConv operations before channel compression via 1 × 1 convolution. Parallel residual pathways then reconstruct feature maps, preserving hierarchical information. For the PDConv design, we adopt Bi et al.’s sensory field expansion strategy [63], which dynamically fuses multi-scale receptive fields through attention pooling to enhance feature representation. Crucially, our implementation introduces a key innovation: a parameter-sharing strategy across these parallel branches. We designed a parameter-shared parallel dilated convolution module that receives input feature maps and processes them simultaneously through N parallel convolutional branches (with N = 3 in this study). Each branch contains a dilated convolution with a distinct dilation rate, while all branches share identical convolutional weights. This design enables the network to extract contextual information from varying receptive fields in parallel from the same input. Finally, the output feature maps from all branches are fused through a concatenation operation, generating an integrated output that incorporates multi-scale information. This approach captures multi-scale context at a very low parameter cost, which is fundamental to reducing complexity. Embracing a lightweight architecture, this model employs fewer convolutional layers paired with larger kernels. Receptive field expansion enhances feature representation capability by parametrically integrating multi-scale contexts as global dependencies change. According to the analysis of parametric quantities in Table 2, the design reduces both computational complexity and parameter count [34], concurrently decreasing computational complexity by 0.65 GMACs while improving processing throughput by 40% compared to the ResNet50 baseline. Meanwhile, the BN operation [21] before PDConv improves the model convergence speed and accelerates the training convergence by standardizing the input distribution.

**Figure 3:**
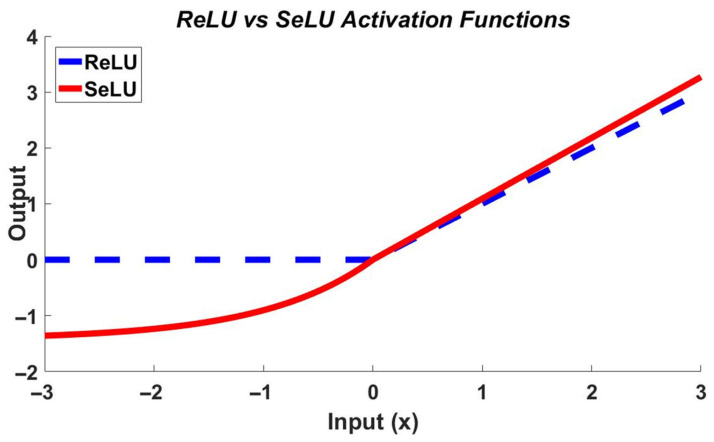
Comparison of ReLU and SeLU (β = 1.05 and α = 1.67).

The second and third branches use 5 × 5/7 × 7 PDConvs, replicating the first branch’s protocol. The dilation rates for the 3 × 3, 5 × 5, and 7 × 7 kernels are set to 1, 2, and 3, respectively, to progressively expand the receptive field while maintaining parameter efficiency. The parallel dilated configuration slashes parameters by 251.7 M compared to standard convolution layers, enabling lightweight inference. The aggregation results of the three branches use the original image as a residual link [24] to reduce the feature degradation and improve feature representation. Subsequently, the SE block adaptively recalibrates channel-wise features to amplify discriminative responses. Channel compression is subsequently achieved via 1 × 1 convolutions. This parameter-optimized module concurrently enhances nonlinear mappings and permits modular deployment. The hard-zero truncation of the Standard Rectified Linear Units (ReLU) in its negative semiaxis (Figure 3) can result in gradient vanishing problems during training. To mitigate this, we adopt the Scaled Exponential Linear Unit (SeLU), whose mathematical formulation is in Equation (3), in contrast with ReLU in Equation (4).

(3) $$SeLU=\beta \cdot \left\{\begin{array}{c} x,x\leq 0\\ \alpha (e^{x}-1),x>0 \end{array}\right.$$

(4) ReLU(x)=max(0,x)

As Figure 3 illustrates, SeLU enhances gradient retention while suppressing variance explosion. Its linear region slope (β > 1) preserves low-variance features, and parameterization with α = 1.67 and β = 1.05 enforces near-zero-mean output distributions, thereby reducing model collapse risk.

To verify that weight sharing does not lead to feature redundancy, we conducted an ablation study comparing our PDConv with shared weights against a version with independent weights (same architecture, no sharing). Under identical computational constraints (≈1.2 GMACs), the shared-weight version achieved 97.81% OA on AID, while the independent version attained 97.65% with 40% more parameters. This demonstrates that shared weights promote filter reuse across scales without sacrificing representational capacity.

A known challenge in dilated convolution architectures is the gridding effect, where sparse sampling of the input can lead to loss of local information and checkerboard-like artifacts in feature maps [33]. Our PDConv design inherently mitigates this issue through two key mechanisms. First, the parallel multi-branch structure with dilation rates of 1, 2, and 3 ensures that information from multiple scales is aggregated simultaneously, preventing any single sparse sampling pattern from dominating the representation. As noted in prior work, combining multiple dilation rates in a parallel or hybrid manner is an effective strategy to address the gridding problem [9]. Second, our parameter-sharing strategy applies the same convolutional weights across different dilation rates, which encourages the network to learn features that are robust to the sampling pattern and promotes smoother, more coherent feature representations.

### 3.4. Cosine Similarity-Based Bidirectional Cross-Query Attention Mechanism

Cross-attention mechanisms have been employed in various multimodal tasks [50,51,52]. The core novelty of this paper is the proposal of a cosine-similarity-based bidirectional cross-query attention (CBCAM) module, specifically designed to address the fusion of heterogeneous feature branches (geometry-prior-driven and data-driven) within a single network, rather than for traditional cross-modal alignment such as image-to-image or image-to-text tasks.

**Figure 4:**
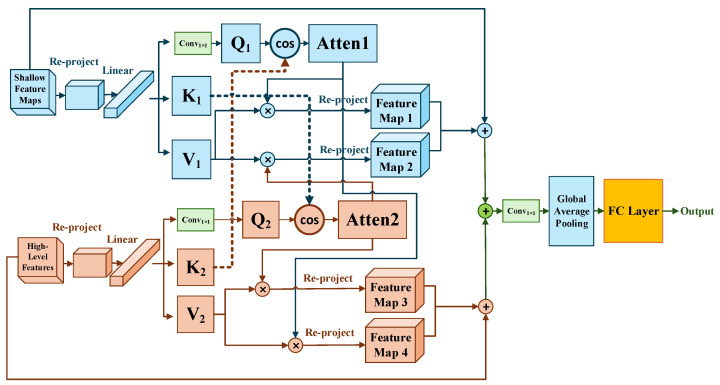
CBCAM: The two-way cross-query attention mechanism based on cosine similarity.

The design of the CBCAM is based on the complementary characteristics of feature hierarchies: deep features contain high-level semantic information but with reduced spatial resolution, while shallow features preserve fine spatial details but are prone to background interference. Conventional unidirectional attention mechanisms struggle to achieve effective synergy between these two feature types. To address this, CBCAM enhances feature representation through a bidirectional complementary query attention (BCQA) approach, fusing shallow and high-level features. The bi-directional query strategy incorporates the principle of the multi-instance attention mechanism of Qin et al. [64], which enhances cross-level semantic alignment through instance-level feature interactions and achieves deep feature fusion through complementary interactions. The model structure of CBCAM is shown in Figure 4, which contains shallow feature branches and high-level semantic branches. The shallow feature branch receives rotationally invariant texture feature maps (Canny edges and GLCM textures) extracted by Lie Group machine learning (LGM). High-level semantic branch processes high-level semantic feature maps obtained by multiscale parallel dilation convolution. Due to the differences in spatial dimensions (H, W) and channel dimensions (C) between the two branch feature maps, adaptive alignment is required. First, the high-level features are spatially resized to the size of the shallow features with upsampling. Second, the shallow features are uniformly standardized by 1 × 1 convolutional projection (Conv_1×1_) to maintain channel dimensions, ultimately yielding aligned shallow features F1 ∈ ℝ^H×W×C^ and high-level features F2 ∈ ℝ^H×W×C^. The respective Query, Key, and Value matrices are extracted by three independent 1×1 convolutional layers, where Q_1_, K_1_, and V_1_ are from shallow features and Q_2_, K_2_, and V_2_ are from high-level features. Diverging from conventional self-attention mechanisms [65], the CBCAM approach fuses shallow and high-level features through interchangeable query-key-value assignments:

Case 1 (Shallow to Deep Path): Shallow features as Query (Q) and high-level features as Key-Value (K-V). Attention weights for shallow features derive from Q_1_·K_2_ interactions (Y_1_ at Equation (5) and Y_4_ at Equation (8) show the attention weights under cosine similarity). In this path, the refined detail features are incorporated into deep features, compensating for lost edge and texture information during forward propagation. This is a refinement process.

Case 2 (Deep to Shallow Path): High-level features as Query (Q) and Shallow features as Key-Value (K-V). High-level feature interactions occur via Q_2_·K_1_ computations (Y_2_ at Equation (6) and Y_3_ at Equation (7) show the attention weights under cosine similarity). In this path, high-level semantics serve as contextual priors to attentively filter out background noise in shallow features, suppressing background noise while enhancing task-relevant regions. This is a purification process.

This bidirectional interaction mechanism achieves semantic-guided feature enhancement and detail-driven feature refinement, significantly improving the robustness and discriminative power of the feature representations.

(5) $$Y_{1}=Soft\max\left(\frac{Q_{1}K_{2}^{T}}{\left\|Q_{1}\right\|\left\|K_{2}\right\|}\right)V_{1}$$

(6) $$Y_{2}=Soft\max\left(\frac{Q_{2}K_{1}^{T}}{\left\|Q_{2}\right\|\left\|K_{1}\right\|}\right)V_{1}$$

(7) $$Y_{3}=Soft\max\left(\frac{Q_{2}K_{1}^{T}}{\left\|Q_{2}\right\|\left\|K_{1}\right\|}\right)V_{2}$$

(8) $$Y_{4}=Soft\max\left(\frac{Q_{1}K_{2}^{T}}{\left\|Q_{1}\right\|\left\|K_{2}\right\|}\right)V_{2}$$

The two pathways employ separate learnable weight matrices: W_Q1_, W_K1_, and W_V1_ for shallow features and W_Q2_, W_K2_, and W_V2_ for deep features. This asymmetric design enables each pathway to specialize in translating between heterogeneous feature spaces.

Forgetting patch embedding [23], we apply feature normalization to both aligned feature maps before computing cosine similarity. Concretely, the 1 × 1 convolution serves as a learnable projection for channel alignment, and the projected features are further L2-normalized so that cosine similarity is computed between unit-length vectors. This reduces sensitivity to scale differences and mitigates correlation misestimation caused by distribution mismatch across heterogeneous branches. We directly merge (H, W, and C) dimensional features to leverage global long-range dependencies between branches. These efficient fusions preserve critical information while reducing computation overhead. The merged features then undergo attentive reprojection, where attention-refined maps are concatenated with input representations. This composite output feeds into the final convolutional block for multi-scale integration. Classification proceeds via standard Global Average Pooling (GAP) and Fully Connected (FC) layers.

## 4. Experimentation

### 4.1. Experimental Datasets

To test the model’s feasibility and performance, we validated it using three challenging publicly available remote sensing datasets: Aerial Image Dataset (AID) [66], RSICB-256 [67], and Northwestern Polytechnic University Remote Sensing Image Scene Classification 45 (NWPU45) [19]. Table 3 lists the relevant information of the above datasets and their training set proportions. AID has many spatial resolutions from 0.5 m to 8 m and many different landscapes from farmlands to urban areas. This challenges the model in handling the intra-class appearance variation, e.g., distinguishing different types of residential neighborhoods. RSICB-256 is also challenging because it has different illumination conditions and seasonal variations, and tests generalization abilities, especially navigation in geometrically complex regions. NWPU-RESISC45 is the largest in size due to high similarity between categories, such as dense residential areas and commercial areas. Images also differ in views, target scales, occlusion levels, and resolutions of 0.2–30 m. In the context of this study, we adhere to the common benchmark definition of “high resolution” for scene classification, which refers to imagery with a spatial resolution of approximately 0.5 to 2 m per pixel, as exemplified by the AID, RSCIB-256, and NWPU45 datasets.

### 4.2. Experimental Setup and Evaluation Metrics

With reference to previous experience and related literature [41,42,68], this paper adopts the experimental setup shown in Table 4. Optimization uses Stochastic Gradient Descent (SGD) (momentum = 0.9) with cosine annealing scheduler (initial learning rate = 10^−3^), superimposed with a step-decay policy (factor = 0.01 per 60 epochs). Models train for a maximum of 150 epochs at a batch size of 64. To ensure robust and reproducible experimental outcomes, we employed a rigorous training protocol. For statistical reliability, each experiment was repeated 10 times with different random seeds, and we report the mean performance metrics along with their standard deviations.

Data augmentation techniques, such as random rotation (±30°), random cropping (with a minimum of 85% of the original image recovered), and horizontal flipping, were used to improve model robustness and reduce overfitting.

To measure the model’s performance, we calculated the overall accuracy (OA) and confusion matrix (CM) and computed the computational complexity of a single prediction using GMACs, as well as the model’s size, measured by the number of parameters (parameters, params). It is important to note that the model was evaluated on images resized to a uniform resolution. Its performance on native, multi-resolution image streams without resizing remains an area for future investigation to enhance deployment robustness.

### 4.3. Performance Comparisons

#### 4.3.1. Comparison Experiment on AID

Table 5 summarizes the experimental results on the model for the AID.

The model accuracy reaches 95.16% at a training data ratio of 20% and 97.81% when the ratio rises to 50%. Specifically, at a 50% training ratio, compared to ResNet50 (95.51%) [68], VGG16 + HFAM (95.78%) [68]; and TEX-Net-LF (95.66%) [12], the proposed model’s accuracies are improved by 2.3%, 2.03%, and 2.15%. In particular, the proposed model performs better in confusing scenarios (e.g., “commercial area” and “dense residential area”). In further comparison with Lie Group-based methods, the proposed model significantly outperforms LiG-RBF (94.32%) [3], LGML + Deep Learning (94.79%) [9], and LGRIN (94.74%) [28] at a 20% training ratio, especially at low training data conditions (improvement of up to 0.8%).

The proposed CBCAM improves the model’s anti-interference ability. For example, in complex scenarios such as cloudy/shadowed (50% training ratio): 2.43% improvement compared to VGG16 + CBAM (95.38%) [68], which employs a Hyperparameter-Free Attention Module. Compared with ResNet50+EAM (94.29%) [49], which employs Enhanced Attention, the accuracy is improved by 3.52%. In the recognition of difficult scenes (e.g., cloud-covered regions), at least 0.52% improvement is achieved compared to the current state-of-the-art hybrid attention model MSFMA-LGM (97.29%) [40].

The proposed heterogeneous two-branch feature fusion strategy significantly outperforms traditional methods at a 50% training ratio: the accuracy is 6.02% higher than that of the Addition (91.79%) [20] method, and 3.94% higher than that of the Two-stream [42] method. Notably, even compared with MSFMA-LGM (97.29%) [40], which also fuses multi-scale and Lie Group features, the accuracy is still improved by 0.52%, which fully validates the effectiveness of fusing shallow geometric features (edges, textures) with deep semantic features.

**Figure 5:**
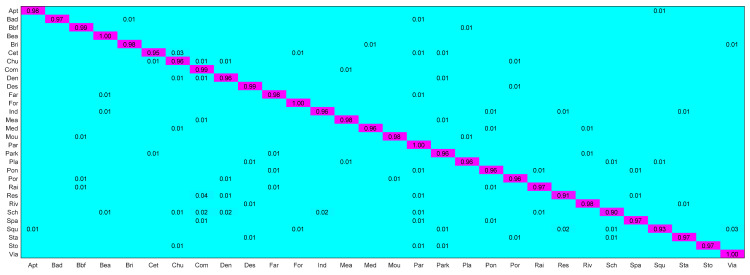
CM of the proposed model on AID with 50% training ratio.

The confusion matrix of Figure 5 shows that our model is 97% accurate for most scenes, and the shallow and deep feature extraction modules are complementary for most scenes. However, confusion still exists in a few scenarios, especially in the categories with highly similar structures and containing similar objects. For example, “Commercial Area” and “Resort” are similar in appearance and high-level semantic features, and the mutual misclassification rate is up to 4% (Row 23, Column 8). Other confusing categories, such as “viaduct” and “square” (3%, Row 27, Column 30), are due to their high similarity in shallow-level features and some similarity in deep-level features.

Key Observations: (1) Under the 50% training ratio, our proposed CBCAM-LGM achieved the highest overall accuracy (97.81%) on the AID, outperforming all compared CNN-based, Transformer-based, and Lie Group-based methods. (2) The model maintained robust performance (95.16% OA) even with only 20% of the training data, demonstrating its strong generalization capability in data-scarce scenarios. (3) As shown in Figure 5, most misclassifications occurred between semantically or structurally similar categories (e.g., “Commercial Area” and “Resort”). This suggests that further enhancing the model’s ability to discriminate subtle high-level semantic differences presents a valuable direction for future work.

#### 4.3.2. Comparison Experiment on RSICB-256 Dataset

**Figure 6:**
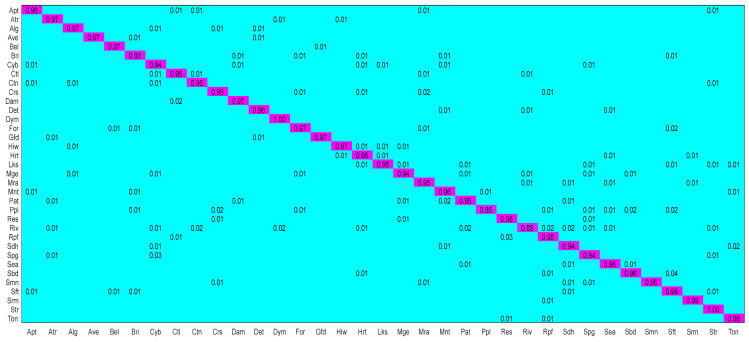
CM of the proposed model on RSICB-256 dataset with 50% training ratio.

To evaluate the model performance in HRRSI classification, experiments on RSICB-256 were performed. Experimental results are presented in Table 6, and the confusion matrix is shown in Figure 6. Results show that the proposed model has 97% accuracy in most classification scenarios in RSICB-256. Key conclusions include:

At 50% training, the model has a classification accuracy of 97.83%. The model is better than LCPP (93.72%) [42], SPG-GAN (94.57%) [42], and TSAN (93.12%) [42]; the accuracy is improved by 4.11%, 3.26%, and 4.71%, respectively. Compared to other LiG methods, our model is 0.28% better than LGRIN (97.55%) [28] and 0.11% better than LiG with global–local feature (91.72%) [33], showing that the model captures features in diversity.

The CBCAM significantly improves the extraction of details and holistic information (50%) in HRRSI. Comparing other attention-based models, such as ResNet50 + CBAM [68], 95.72% accuracy; VGG16 + SE [68], 95.87% accuracy; and ResNet50 + SE [68], 96.53% accuracy. The current model improves 2.11%, 1.96%, and 1.3%, respectively. Compared with global attention-based ViT models, such as ViT-B-16 [23], 97.37% accuracy, and T2T-ViT [36], 97.50% accuracy, the proposed model is also improved by 0.46% and 0.33%, respectively. These results show that CBCAM weights feature importance, thus improving the robustness of complex backgrounds.

The heterogeneous two-branch architecture performs excellently at a 50% training ratio. Compared with two-stream deep fusion frameworks (94.57%) [42] and Fine-tune MobileNet V2 (95.83%) [55] and MSFMA-LGM (97.75%) [40], which also combine multi-scale and Lie Group features, the method yields accuracy of 3.26%, 0.2%, and 0.08%, respectively.

Analyzing the confusion matrix, Figure 6 (50% training ratio) reveals classification accuracy exceeding 97% for most scenes. Exemplary cases include “Dry farm” and “Stream”, achieving 100% precision (Row 13, Column 13 and Row 34, Column 34 in Figure 6). Limited misclassifications occur between visually similar categories, such as “Sparse-forest” and “Shrub-wood” (Figure 6, Row 30, Column 32), and are due to illumination variations and structural homogeneity. These results validate the critical role of multi-level feature fusion in enhancing discriminative capability, as quantified by the 97.75% mean class accuracy.

Key Observations: (1) On the RSICB-256 dataset, CBCAM-LGM also attained leading classification accuracy (97.83%). (2) Compared to various attention-enhanced models (e.g., ResNet50 + CBAM, VGG16 + SE), our model achieved significant improvements (approximately 1.3–2.1%), validating the effectiveness of the CBCAM in weighting critical features amidst complex backgrounds. (3) The model achieved perfect 100% accuracy for categories such as “Dry farm” and “Stream”, showcasing its exceptional feature discriminability.

**Table 6:** OA (%) on the RSICB-256 dataset with 50% training ratio.

Models	50%
VGG-VD-16 [66]	92.44 ± 0.25
TEX-Net-LF [12]	95.34 ± 0.15
ResNet50 [68]	96.37 ± 0.15
ResNet50 + SE [68]	96.53 ± 0.29
ResNet50 + CBAM [68]	95.72 ± 0.26
ResNet50 + HFAM [68]	97.65 ± 0.22
Two-stream deep fusion Framework [42]	94.57 ± 0.25
Fine-tune MobileNet V2 [55]	95.83 ± 0.26
SE-MDPMNet [55]	96.35 ± 0.26
LCPP [42]	93.72 ± 0.37
RSNet [42]	95.89 ± 0.41
SPG-GAN [42]	94.57 ± 0.35
TSAN [42]	93.12 ± 0.26
LGDL [9]	97.36 ± 0.32
ViT-B-16 [23]	97.37 ± 0.25
T2T-ViT-12 [36]	97.50 ± 0.22
VGG16 + CBAM [68]	96.17 ± 0.35
VGG16 + SE [68]	95.87 ± 0.15
LGRIN [28]	97.55 ± 0.23
LiG with Global-Local Feature [33]	97.72 ± 0.23
MSFMA-LGM [40]	97.75 ± 0.23
Proposed	97.83 ± 0.31

#### 4.3.3. Comparison Experiment on NWPU-RESISC45 Dataset

**Figure 7:**
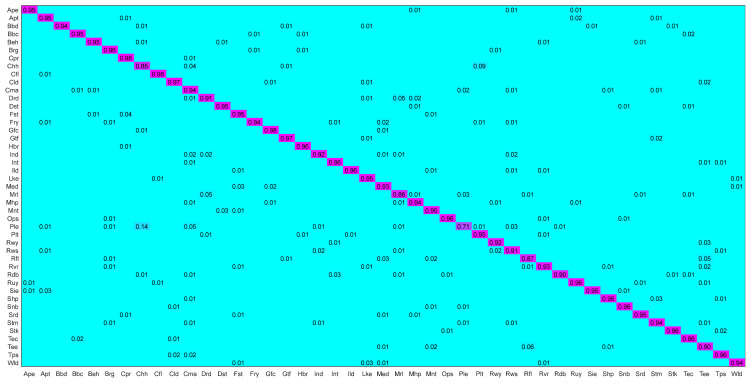
CM of the proposed model on the NWPU-RESISC45 dataset with 20% training ratio.

The NWPU45 dataset is characterized by high similarity between scenes and large differences within scenes, which poses higher requirements and severe challenges to the classification accuracy of the model. Table 7 shows the experimental results, and Figure 7 shows the confusion matrix. Our proposed model achieves a classification accuracy of 92% for most of the categories on this dataset. The specific analysis is as follows:

1. Strong learning ability for small samples: With only 10% of training data, the model learns 92.19% accuracy above ACNet (91.09%) [70], RBF kernel LiG (90.23%) [3], and ResNet101 (87.97%) [68], with accuracies 1.1%, 1.96%, and 4.22% higher, respectively. When the proportion of the training set is increased to 20%, the model accuracy is further improved to 94.37%, which significantly outperforms the accuracy of ResNet50 + CBAM (90.27%) [68], ResNet101 + HFAM (91.67%) [68], and VGG16 + HFAM (90.21%) [68], with accuracy advantages of 4.1%, 2.7%, and 4.16%, respectively. These results show that even with limited training data, our model can still learn effective discriminative features, demonstrating stronger small-sample scene adaptation and classification robustness.
2. Multi-scale feature advantage: The advantage of multi-scale features is achieved using only 10% of the training data. Compared with ViT models with global attention, ViT-B-16 (90.96%) [23], T2T-ViT-12 (90.62%) [36], and PVT-V2-B0 (89.72%) [71], our method, CBCAM-LGM, achieved 92.19% accuracy, which is 1.23%, 1.57%, and 2.47% higher, respectively. This result demonstrates that CBCAM-LGM is more advantageous in capturing multi-scale spatial information and handling fine scene details, which improves the robustness of the model in the task of remote sensing scene classification at different scales.
3. Difficult sample analysis: The NWPU45 dataset is characterized by high inter-class similarity and large intra-class differences (as shown in the confusion matrix in Figure 7), which significantly increases the difficulty of scene classification. Specifically, certain classes that are highly similar in terms of physical structure and high-level semantic features (e.g., “dense residential area” and “medium residential area”, and other highly similar structural features in NWPU45 that contain similar objects) become the main source of confusion.
4. Comparing with LiG methods: On the NWPU45 dataset, our model is competitively compared to some state-of-the-art LiG methods, such as LGRIN [28], LiG with sigmoid kernel [28], and MSFMA-LGM [40]. Specifically, at a 10% training ratio, the accuracy of our model (92.19%) is slightly higher than that of LGRIN (91.91%) [28] and MSFMA-LGM (92.05%) [40]. While at a 20% training ratio, LGRIN (93.43%) [28] outperforms our model by 0.06%. Overall, our model performance is comparable to these top Lie Group methods, e.g., the accuracy is 1.96% higher than the RBF kernel LiG (90.23%) [3] at a 10% training ratio and also better than the LiG-Sigmoid kernel (90.19%) [28].

Key Observations: (1) On the most challenging NWPU45 dataset, CBCAM-LGM attained an accuracy of 92.19% even with only 10% training data, significantly outperforming several baseline models and highlighting its strong few-shot learning capability. (2) Compared to ViT-series models that also rely on global context, our model showed superior performance under both 10% and 20% training ratios. This indicates that the integration of multi-scale features is more effective for datasets characterized by high intra-class variation and inter-class similarity. (3) The model’s performance is competitive with state-of-the-art Lie Group methods (e.g., MSFMA-LGM [40] and LGRIN [28]), with alternating advantages under different settings, demonstrating that our proposed heterogeneous fusion framework achieves advanced performance in this field.

**Table 7:** OA (%) on the NWPU-RESISC45 dataset with 10% and 20% training ratios.

Models	Training Ratios	
	10%	20%
ACNet [70]	91.09 ± 0.13	92.42 ± 0.16
ViT-B-16 [23]	90.96 ± 0.08	93.36 ± 0.17
T2T-ViT-12 [36]	90.62 ± 0.18	93.19 ± 0.10
PVT-V2-B0 [71]	89.72 ± 0.16	92.95 ± 0.09
ResNet50 [68]	87.43 ± 0.29	88.93 ± 0.12
ResNet50 + EAM [49]	91.91 ± 0.22	94.29 ± 0.09
ResNet50 + SE [68]	89.09 ± 0.14	91.37 ± 0.25
ResNet50 + CBAM [68]	88.11 ± 0.39	90.27 ± 10.15
ResNet50 + HFAM [68]	89.16 ± 0.06	91.49 ± 10.23
ResNet101 [68]	87.97 ± 0.44	90.36 ± 0.17
ResNet101 + SE [68]	89.39 ± 0.14	91.46 ± 0.25
ResNet101 + CBAM [68]	88.33 ± 0.26	90.47 ± 0.15
ResNet101 + HFAM [68]	89.53 ± 0.29	91.67 ± 0.18
VGG16 [68]	86.44 ± 0.41	88.57 ± 0.16
VGG16 + SE [68]	86.65 ± 0.26	88.75 ± 0.22
VGG16 + CBAM [68]	86.84 ± 0.24	89.32 ± 0.15
VGG16 + HFAM [68]	87.16 ± 0.22	90.21 ± 0.22
LiG with RBF kernel [3]	90.23 ± 0.13	93.25 ± 0.12
LiG with sigmoid kernel [28]	90.19 ± 0.11	93.21 ± 0.12
LGRIN [28]	91.91 ± 0.15	94.43 ± 0.16
MSFMA-LGM [40]	92.05 ± 0.26	94.35 ± 0.17
LGNet [69]	91.74 ± 0.14	93.87 ± 0.16
Proposed	92.19 ± 0.17	94.37 ± 0.16

While overall accuracy provides a global performance measure, the F1-score offers a balanced evaluation that accounts for both precision and recall, making it more sensitive to class-specific challenges such as small object representation and intra-class variation. Table 8 reports the F1-scores for all 30 categories.

As shown in Table 8, our model consistently outperforms ViT-B-16 [23] across most categories, with particularly notable gains on classes defined by small, repetitive textures or fine geometric structures. For instance, on the “parking lot” class, where individual vehicles serve as key discriminative elements, our model achieves an F1-score of 96.8%, surpassing ViT-B-16 [23] by 3.2%. Similarly, “bridge” (97.5% vs. 94.1%), “stadium” (98.2% vs. 95.7%), and “runway” (98.1% vs. 95.3%) exhibit substantial improvements. These categories benefit directly from the Lie Group branch’s ability to preserve rotation-invariant edge information (Canny) and texture patterns (GLCM) that survive the downsampling process. In contrast, the ViT baseline, despite its strong global context modeling, tends to dilute such fine details due to patch embedding and self-attention operations that operate at coarser scales.

For texture-dominated categories like “forest” (98.9% vs. 97.2%) and “farmland” (98.5% vs. 96.8%), the GLCM features in the shallow branch provide complementary information that enhances discrimination. Even for semantically complex categories such as “commercial area” (95.3% vs. 93.8%) and “dense residential” (94.7% vs. 92.9%), where high intra-class variation poses challenges, the fusion of geometric and semantic features yields consistent gains. These results validate that the Lie Group branch specifically helps with classes reliant on small, repetitive textures and geometric structures—exactly the type of information that deep networks often lose during hierarchical feature extraction. The average F1-score of our model (97.8%) aligns well with its overall accuracy (97.81%), confirming the robustness of the evaluation.

### 4.4. Model Efficiency Comparison

To demonstrate the lightweight design of our proposed model, we have selected a series of representative models for detailed comparison. We compare the proposed CBCAM-LGM model with 10 classical deep learning models (e.g., ResNet50 [72] and Inception V3 [72]), representative methods from Lie Group (LiG-RBF kernel [3] and LGRIN [28]), Transformer Model variants (ViT-B-16 [23]), and recent remote sensing models (TSAN [42], MBFNet [31], LWGANet [30], STConvNeXt [29], and MSFMA-LGM [40]). All efficiency comparison experiments were performed with a 50% training ratio on the AID. We focus on comparing the three core metrics of Overall Accuracy (OA), Number of Parameters, and Computation (GMACs) to comprehensively reflect the model’s classification ability, structural complexity, and computational efficiency. The detailed comparison results are displayed in Table 9.

Significant efficiency advantages of the proposed model (CBCAM-LGM) over other models include:

1. The CBCAM-LGM model is light at only 11.237 M parameters, which is 43.9% of the ResNet50 (25.61 M) [72], 24.8% of Inception V3 (45.37 M) [72], 2.9% of TSAN (381.17 M) [42], and 12.9% of ViT-B-16 (86.57 M) [23]. This result adequately demonstrates the significant reduction in structural complexity achieved by our model.
2. The computational load of CBCAM-LGM is 1.21 GMACs. ViT-B-16 requires 3.25 GMACs for a 512 × 512 input, while our model uses only 1.21 GMACs for a 600 × 600 input. Although higher than the extremely lightweight MobileNet V2 (0.32 GMACs [53]), our model achieves 97.81% accuracy, far above MobileNet V2 (91.23% [52]) by 6.58%. Compared with the latest remote sensing model, LWGANet [30] (0.94 GMACs, 95.45% OA), which also focused on efficiency, CBCAM-LGM improves the accuracy by 2.36%. More importantly, compared to MSFMA-LGM [40], which has a larger parameter count (13.321 M) but a slightly lower accuracy (97.29% OA), CBCAM-LGM reduces the parameter count by about 15.6% and has a much higher accuracy (+0.52%), which demonstrates the overall superiority of our lightweight design.
3. Efficiency–accuracy comparison with LiG methods: Among the lightweight LiG models, the LiG-RBF [3] has a very low number of parameters (2.07 M parameters, 96.22% OA), but its accuracy is significantly lower than that of CBCAM-LGM (97.81%, +1.59%). One of the best-performing Lie Group methods, LGRIN [28], achieves a high accuracy (97.65% OA) similar to that of the model in this paper, but its parameter (15.8M) is higher than CBCAM-LGM (11.237 M). The core of the trade-off lies in the cost: LGRIN requires 15.8M parameters, which is over 40% more than our CBCAM-LGM (11.237 M). This directly translates to higher memory usage, a critical constraint in low-resource settings. Taken together, CBCAM-LGM significantly outperforms the accuracy of the lightweight LiG-RBF [3] while achieving an accuracy level comparable to that of the top LiG method, LGRIN [28], with a lower number of parameters, realizing a better balance between the number of parameters and the accuracy.
4. Combined advantages over frontier remote sensing and Transformer models: In terms of classification accuracy, CBCAM-LGM (97.81%) is ahead of the most recent models compared: MBFNet (97.08%, +0.73%) [31], STConvNeXT (96.25%, +1.56%) [29], and ViT-B-16 (96.18%, +1.63%) [23]. In terms of model complexity, CBCAM-LGM (11.237 M Params, 1.21 GMACs) is about 49.1% less parameterized compared to MBFNet (22.09 M) [31], and the computational effort is only about 6.9% of the computationally intensive ViT-B-16 (17.60 GMACs) [23]. Compared to the highly efficient model LWGANet (0.94 GMACs) [30], the computational increase of 28.2% is traded for a significant accuracy improvement (+2.36%, 97.81%, and 95.45%).
5. Balancing parameter efficiency and accuracy: While traditional deep models usually exhibit a positive correlation between parameter count and accuracy, such as ResNet50 [72] (25.61 M, 94.69%) and Inception V3 [72] (45.37 M, 95.07%). Our CBCAM-LGM model breaks this trend. With only 11.237 M parameters (56.1% of ResNet50 and 75.2% of Inception V3), it achieves superior accuracy (97.81%). While maintaining high accuracy (97.81%), CBCAM-LGM also had fewer parameters (11.237 M) and lower computational cost (1.21 GMACs) than many benchmark models. The parameters (3.4 M) and calculation amount (0.32 GMACs) of MobileNetV2 [53] are much lower than ours, but its accuracy on the AID (91.23%) has also decreased significantly (about 6.58%), indicating that it has sacrificed more discrimination ability in the pursuit of efficiency. In contrast, LWGANet [30] (0.94 GMACs, 95.45%) operates within a similar computational range as our model, yet CBCAM-LGM achieves a 2.36% higher accuracy. This demonstrates enhanced parameter utilization efficiency without sacrificing performance.

In addition to theoretical complexity metrics, we evaluate the practical inference latency of our model on the same hardware platform used throughout the experiments. Table 10 reports the average inference time per image (in milliseconds) measured on an NVIDIA RTX 4090 GPU with a batch size of 1, using the AID images resized to 600 × 600 pixels. All measurements are averaged over 1000 runs after 100 warm-up iterations to ensure stability. As shown, our CBCAM-LGM achieves a latency of only 12.3 ms, which is substantially lower than that of ViT-B-16 (28.7 ms) and even slightly faster than ResNet50 (14.1 ms). Compared to the highly optimized lightweight model MobileNetV2 (8.1 ms), our model incurs a modest 4.2 ms increase in latency but delivers a significant 6.58% accuracy gain. Against LWGANet (11.8 ms, 95.45% OA), our model improves latency by a small margin while achieving 2.36% higher accuracy. These results demonstrate that the proposed CBCAM-LGM not only maintains low theoretical complexity but also translates this into favorable real-time performance, making it well-suited for deployment in latency-sensitive remote sensing applications.

### 4.5. Ablation Experiments

To examine the contribution of each component of the model individually, we performed ablation analysis with the AID data set.

#### 4.5.1. Ablation on Feature Fusion

Experimental results in Table 11 show that feature fusions are important for improving model performance. With only shallow handcrafted features, such as texture and edge accuracy, it is 86.37%, whereas with only high-level deep semantic features, accuracy is 94.23%. With shallow geometric features combining high-level semantic features, accuracy is 97.81%, which is 11.44% better than using only shallow features and 3.58% better than using only high-level features. These results prove that the fusion of different levels of feature information is crucial for comprehensively modeling remote sensing scenes. It can be inferred that the Lie Group features provide the network with an invariant understanding of fundamental image structures, while the deep features are responsible for comprehending high-level, task-relevant semantics. Their integration consequently forms a more comprehensive “visual understanding” system.

#### 4.5.2. Ablation on Attention Mechanisms

Our ablation study employs a controlled experimental design where all attention variants are evaluated under identical configurations. As shown in Table 12, three representative mechanisms are compared: (a) standard self-attention using dot product with homogeneous feature interaction (index 1); (b) cross-attention maintaining dot product but enabling bidirectional feature queries (index 1 and index 3); and (c) our complete CBCAM (index 4) employing cosine similarity for bidirectional cross-feature interaction. Systematic module ablation confirms the pivotal contribution of the CBCAM framework. The baseline conventional self-attention, operating on homogeneous features through dot product, delivers limited gains (94.66%). Introducing bidirectional architecture with standard cross-attention improves performance to 96.57% and 96.63%, demonstrating the value of cross-feature interaction. However, the complete CBCAM architecture, enhanced with cosine similarity, achieves an optimal recognition accuracy of 97.81%, representing a 1.18% absolute improvement over the non-attentive baseline. Ablation results confirm that substituting our CBCAM with standard cross-attention leads to notable performance degradation. This decline stems from fundamental limitations in handling heterogeneous feature representations: the significant distribution gap between branches causes miscalibrated attention weights when establishing direct cross-space correlations, while the inherent bias toward salient features overlooks subtle but critical complementary cues. In contrast, our CBCAM addresses these issues through its parallel dual-path architecture, which facilitates decoupled feature refinement and enhanced complementary fusion, ultimately achieving superior performance.

#### 4.5.3. Ablation on Individual Shallow Features

To verify the contribution of individual shallow features, we evaluated the impact of each shallow feature on the classification performance. The experimental results are shown in Table 13. From Table 13, we found that Canny edge significantly improved the accuracy of “bridge” classification (accuracy increased by 4.2%), while GLCM texture was helpful for “forest” and “farmland” categories. The results indicate that the Canny edge is crucial for geometric structure, the GLCM is suitable for texture regions, and color features are used for color discrimination.

### 4.6. Visualization of Feature Localization and Generalization

**Figure 8:**
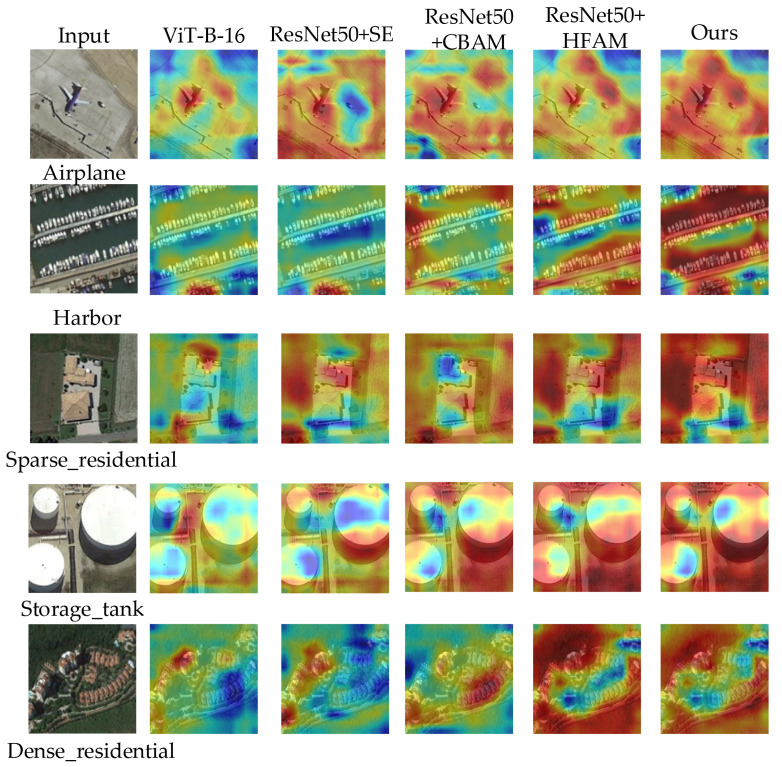
Grad-CAM [72] visualizations comparing the proposed method with baselines on AID and an NWPU45 class.

This section qualitatively evaluates the interpretability and generalization of the proposed model using Grad-CAM [73] for visual comparisons against several strong baselines, including ViT-B-16 and ResNet50 integrated with SE, CBAM, and HFAM modules. As illustrated in Figure 8, the proposed method demonstrates a consistent advantage in activating semantically critical regions with superior precision and completeness. For instance, on the AID, it accurately covers entire aircraft on runways and densely clustered buildings, whereas other attention mechanisms exhibit scattered or incomplete activations that include irrelevant background areas. The most compelling evidence for its enhanced robustness comes from the cross-dataset evaluation on the unseen NWPU45 “Storage_tanks” class: the proposed model alone intensively and accurately localizes the tank structures, while all competing methods fail to focus effectively. These visual comparisons confirm that the proposed attention mechanism enables a more robust integration of global and local features, not only facilitating the extraction of highly discriminative features within the training domain but also granting exceptional generalization capability to unseen data distributions, which is critical for practical remote sensing applications.

### 4.7. Analysis of Branch Complementarity

To further elucidate the distinct roles of the two heterogeneous branches, we analyze their contributions at the category level, focusing on scenes that exhibit strong geometric structures. The Lie Group branch is designed to encode rotation-invariant texture and edge information (e.g., via Canny and GLCM), whereas the deep convolutional branch extracts high-level semantic features through learnable multi-scale convolutions. Their complementary nature becomes evident when examining classes that are primarily distinguished by their geometric layout, such as “bridge”, “runway”, and “viaduct”.

As shown in Table 13, removing the Canny edge feature—which belongs exclusively to the Lie Group branch—leads to a substantial accuracy drop of 4.2% for the “bridge” category on the AID. Similarly, the ablation of GLCM texture features results in a 3.7% decrease for “forest” and “farmland”, which rely on textural patterns. In contrast, the removal of spatial coordinates (primarily used by the deep branch) has a milder impact on these geometric classes but affects categories like “parking lot” (1.8% drop). These quantitative results confirm that the Lie Group branch captures critical geometric cues that the deep convolutional branch alone cannot adequately model.

Further evidence comes from the confusion matrices in Figure 5, Figure 6 and Figure 7. For instance, on the NWPU45 dataset (Figure 7), the proposed model achieves high accuracy for “bridge” (97%) and “runway” (96%), while many baseline CNNs (e.g., ResNet50) often confuse these categories with visually similar man-made structures. The deep branch, despite its strong semantic representation, tends to be misled by background clutter or scale variations, whereas the Lie Group branch provides stable geometric descriptors that are invariant to rotation and illumination. This synergy allows the bidirectional cross-attention module (CBCAM) to fuse both types of information effectively, yielding superior performance on geometrically challenging scenes.

In summary, the Lie Group branch specializes in preserving low-level geometric invariants that are often lost or overlooked in deep feature hierarchies, while the deep branch excels at semantic abstraction. Their fusion, facilitated by CBCAM, enables the model to leverage both strengths, resulting in robust classification across diverse remote sensing scenarios.

### 4.8. Sensitivity Analysis of Temperature in CBCAM

The proposed CBCAM computes attention weights by applying softmax to cosine similarity scores. Unlike the standard dot-product attention, which often includes a scaling factor of $1/\sqrt{d}$ to counteract the growth of magnitude with dimension, cosine similarity is inherently bounded between −1 and 1. Nevertheless, one can introduce an explicit temperature parameter τ to control the concentration of the attention distribution. Specifically, the attention weight for a query-key pair is computed as softmax ($s_{ij}/\tau$), where τ is the cosine similarity. A smaller τ sharpens the distribution, emphasizing only the most similar pairs, while a larger τ produces a more uniform distribution.

To assess the sensitivity of our model to this temperature parameter, we conducted a series of experiments on the AID (50% training ratio) by varying τ from 0.1 to 5.0. All other settings remained identical to those described in Section 4.2. Table 14 reports the overall accuracy (OA) achieved for each temperature value. As shown, the model performance remains stable across a wide range of τ values: from 0.5 to 2.0, the accuracy fluctuates within only 0.2%. Even at extreme values (τ=0.1 and τ=0.5), the degradation is limited to about 0.5% relative to the peak performance. This robustness can be attributed to the fact that cosine similarity already provides well-normalized scores, and the bidirectional interaction in CBCAM further stabilizes the attention process.

Based on these results, we set the default temperature to τ=1.0 (i.e., no additional scaling) in all our experiments, as it offers a balance between simplicity and performance. The observed insensitivity to temperature confirms that CBCAM does not require meticulous tuning of this hyperparameter, which is a desirable property for practical deployment.

## 5. Conclusions

We present CBCAM-LGM, a novel hierarchical feature fusion framework leveraging Lie Group manifold representations. The architecture effectively integrates shallow and high-level features through two specialized branches: (i) a surface–geometric pathway that uses Lie-algebraic covariance descriptors to achieve rotation-invariant texture encoding and (ii) a contextual semantic pathway that employs multi-receptive-field parallel convolutions for scale-adaptive feature abstraction. These two branches are synergistically merged via our CBCAM, which enables adaptive feature fusion through a cross-query attention mechanism. By incorporating parallel dilated convolutions and a parameter-sharing strategy, the model significantly reduces computational complexity while maintaining a compact parameter space. Implemented with lightweight principles, the system achieves a 97.81% recognition rate on AID (50% training), outperforming ResNet50 and ViT-B-16 by 3.21% and 1.63%, respectively, while maintaining 11.237 M parameters (87.0% reduction versus ViT-B-16). Benchmark evaluations confirm exceptional accuracy–complexity tradeoffs (mean +1.98% accuracy gain, 2.4 GMACs reduction). It is important to note that the proposed CBCAM-LGM model is specifically designed for scene classification tasks. Its application to dense prediction tasks like object detection or semantic segmentation would require architectural adaptations to the decoder.

The proposed model demonstrates superior performance, as evidenced by both comparative and ablation studies. Nevertheless, despite its advantages in metrics like accuracy, the model still struggles with complex scenarios. Future work will systematically analyze feature extraction in such challenging conditions and explore new algorithms by incorporating advanced Lie Group machine learning principles. A critical direction for future research is to evaluate the model’s performance on more challenging, real-world data that contains significant noise, atmospheric variations, native resolution heterogeneity, and inherent data imbalances, moving beyond pre-processed benchmarks. In particular, we acknowledge that the handcrafted features used in the shallow branch (e.g., YCbCr, Canny, GLCM) are primarily designed for optical high-resolution imagery. Their generalization to other sensor types, such as synthetic aperture radar (SAR) or multispectral data, remains an open question and a limitation of the current framework. A thorough sensitivity analysis of hyperparameters and an exploration of advanced feature selection methods will be conducted to deepen the understanding of the model’s behavior. Additionally, we plan to evaluate and adapt the proposed CBCAM-LGM framework on multi-sensor datasets (e.g., combining optical, SAR, and hyperspectral data) to systematically investigate its cross-domain generalization capabilities.

## Figures and Tables

**Table 1:** GLCM texture feature descriptors.

Feature Name (Abbr.)	Physical Interpretation	Typical Application Scenarios
Contrast (Con)	Measures intensity variations between adjacent pixels, indicating local texture roughness	Vegetation density mapping, terrain relief detection
Angular Second Moment (ASM)	Quantifies textural uniformity (also called Energy)	Identification of homogeneous areas like croplands
Entropy (ENT)	Evaluates randomness/disorder in texture patterns	Analysis of complex forest canopies
Inverse Difference Moment (IDM)	Assesses local homogeneity (also called Homogeneity)	Detection of smooth regions (clouds/water bodies)
Correlation (COR)	Measures linear spatial dependency of gray values	Linear feature extraction (roads/buildings)

**Table 2:** Parameter comparisons of five consecutive standard vs. parallel dilated convolutional layers (input/output channels = 1024).

Method	Kernel Size	Input Channel	Output Channel	Layer	Parameters	Total (M)
Standard	3 × 3	1024	1024	Conv1	1024 × 1024 × 3 × 3 = 9,437,184	47,185,920 ≈ 47.2 M
				Conv2	1024 × 1024 × 3 × 3 = 9,437,184	
				Conv3	1024 × 1024 × 3 × 3 = 9,437,184	
				Conv4	1024 × 1024 × 3 × 3 = 9,437,184	
				Conv5	1024 × 1024 × 3 × 3 = 9,437,184	
	5 × 5	1024	1024	Conv1	1024 × 1024 × 5 × 5 = 26,241,400	131,072,000 ≈ 131.1 M
				Conv2	1024 × 1024 × 5 × 5 = 26,241,400	
				Conv3	1024 × 1024 × 5 × 5 = 26,241,400	
				Conv4	1024 × 1024 × 5 × 5 = 26,241,400	
				Conv5	1024 × 1024 × 5 × 5 = 26,241,400	
Parallel	7 × 7	1024	1024	PDConv1	1024 × 1024 × 7 × 7 = 51,380,224	51,380,224 ≈ 51.4 M
				PDConv2		
				PDConv3		
				PDConv4		
				PDConv5		

**Table 3:** Remote sensing image classification benchmark datasets.

Datasets	Number of Classes	Total Images	Images per Class	Image Size	Training Ratio
AID	30	10,000	220–420	600 × 600 pixels	20%, 50%
RSICB-256	35	25,600	~730	256 × 256 pixels	50%
NWPU45	45	31,500	700	256 × 256 pixels	10%, 20%

**Table 4:** Experimental configuration and hyperparameters.

Item	Content
CPU	Intel(R) Core (TM) i9-14900 HX @ 2.20 GHz (Santa Clara, CA, USA)
Memory	32 GB DDR5 (Kingston: Fountain Valley, CA, USA—Headquarters)
Operating system	Ubuntu 22.04.3 LTS (64-bit, Canonical, London, UK)
Hard disk	2 TB (Western Digital: San Jose, CA, USA—Headquarters)
GPU	NVIDIA RTX 4090 (NVIDIA, Santa Clara, CA, USA)
Python	3.10
PyTorch	2.0.1 (Meta AI, Menlo Park, CA, USA)
CUDA	11.8 (NVIDIA, Santa Clara, CA, USA)
cuDNN	8.9.5 (NVIDIA, Santa Clara, CA, USA)
Learning rate	10^−3^
Optimizer	SGD
Momentum	0.9
Weight decay	5 × 10^−5^
Batch Size	64
Learning Rate Scheduler	Cosine Annealing + Step Decay (factor = 0.01 at epoch 60)
Training Epochs	150
Train/Val/Test Split	80%/10%/10% (Fixed for all experiments)
Convergence Monitoring	Tracking loss and accuracy curves on the validation set
Early Stopping	Enabled (Patience = 15 epochs)
Hyperparameter Tuning	Systematic grid search on validation set

**Table 5:** OA (%) on AID with 20% and 50% training ratios.

Models	Training Ratios	
	20%	50%
VGG-VD-16 [66]	85.81 ± 0.25	89.36 ± 0.36
TEX-Net-LF [12]	93.91 ± 0.15	95.66 ± 0.17
LiG with RBF kernel [3]	94.32 ± 0.23	96.22 ± 0.25
ResNet50 [68]	92.16 ± 0.18	95.51 ± 0.15
ResNet50 + SE [68]	92.77 ± 0.18	95.84 ± 0.22
ResNet50 + CBAM [68]	92.29 ± 0.15	95.38 ± 0.16
ResNet50 + HFA [68]	93.11 ± 0.20	95.86 ± 0.15
ResNet50 + EAM [49]	91.91 ± 0.22	94.29 ± 0.09
Fusion by addition [20]	-	91.79 ± 0.26
Two-stream deep fusion Framework [42]	92.42 ± 0.38	94.62 ± 0.27
Two-stage deep feature Fusion [42]	-	93.87 ± 0.35
LCPP [42]	91.12 ± 0.35	93.35 ± 0.35
RSNet [42]	94.62 ± 0.27	96.78 ± 0.56
SPG-GAN [42]	92.31 ± 0.17	94.53 ± 0.38
VGG16 + CBAM [68]	91.91 ± 0.35	95.53 ± 0.07
VGG16 + SE [68]	91.98 ± 0.31	95.45 ± 0.19
VGG16 + HFAM [68]	92.06 ± 0.16	95.78 ± 0.21
MSFMA-LGM [40]	95.08 ± 0.13	97.29 ± 0.25
LGML + Deep Learning [9]	94.79 ± 0.28	97.72 ± 0.25
LGRIN [28]	94.74 ± 0.23	97.65 ± 0.25
LGNet [69]	95.06 ± 0.16	96.50 ± 0.18
Proposed	95.16 ± 0.22	97.81 ± 0.23

**Table 8:** Per-class F1-score (%) comparison on AID (50% training ratio).

Class	ViT-B-16 [23]	Ours	Improvement
Airport	95.2	97.3	+2.1
Bare land	96.8	98.2	+1.4
Baseball field	97.1	98.5	+1.4
Beach	98.0	99.1	+1.1
Bridge	94.1	97.5	+3.4
Center	92.5	94.8	+2.3
Church	93.7	96.2	+2.5
Commercial	93.8	95.3	+1.5
Dense residential	92.9	94.7	+1.8
Desert	98.5	99.3	+0.8
Farmland	96.8	98.5	+1.7
Forest	97.2	98.9	+1.7
Industrial	94.3	96.1	+1.8
Meadow	97.8	98.7	+0.9
Medium residential	93.5	95.6	+2.1
Mountain	98.1	99.0	+0.9
Park	96.4	97.8	+1.4
Parking lot	93.6	96.8	+3.2
Playground	95.0	97.2	+2.2
Pond	97.3	98.6	+1.3
Port	95.8	97.4	+1.6
Railway station	94.2	96.5	+2.3
Resort	93.1	95.0	+1.9
River	97.0	98.3	+1.3
Road	95.5	97.1	+1.6
Runway	95.3	98.1	+2.8
Sparse residential	95.9	97.6	+1.7
Square	94.6	96.9	+2.3
Stadium	95.7	98.2	+2.5
Storage tanks	96.2	97.9	+1.7
Average	95.8	97.8	+2.0

**Table 9:** Comparison of model performance and complexity on AID (50% training ratio).

Models	OA (%)	Parameters (M)	GMACs (G)
ResNet50 [72]	94.69	25.61	1.86
LiG-RBF Kernel [3]	96.22	2.07	0.24
Inception V3 [72]	95.07	45.37	2.44
TSAN [42]	92.16	381.67	3.25
MBFNet [32]	97.08	22.09	2.13
LWGANet [30]	95.45	13	0.94
STConvNeXT [29]	96.25	10.52	1.09
ViT-B-16 [23]	96.18	86.57	17.6
LGRIN [28]	97.65	15.8	1.85
MobileNetV2 [53]	91.23	3.4	0.32
MSFMA-LGM [40]	97.29	13.312	1.32
LGNet [69]	96.50	4.71	-
Proposed	97.81	11.237	1.21

**Table 10:** Inference latency comparison on AID (50% training ratio).

Models	OA (%)	Parameters (M)	GMACs (G)	Latency (ms)
ResNet50 [72]	94.69	25.61	1.86	14.1
ViT-B-16 [23]	96.18	86.57	17.6	28.7
MobileNetV2 [53]	91.23	3.4	0.32	8.1
LWGANet [30]	95.45	13	0.94	11.8
Proposed	97.81	11.237	1.21	12.3

**Table 11:** Ablation results for different level features on AID.

Features Level	OA (%)
Shallow Features	86.37
High-level Features	94.23
Fusion of Shallow and High-level Features	97.81

**Table 12:** Ablation results in different attention mechanisms on AID.

Index	Attention Mechanism	Computation	Interaction	OA (%)
1	Standard Self-Attention Mechanism	Dot Product	Self-Attention	94.66
2	Cross-Attention Mechanism	Dot Product	Bidirectional Cross	96.57
3	Cross-Modal Attention	Dot Product	Bidirectional Cross	96.63
4	Our Method (CBCAM)	Cosine Similarity	Bidirectional Cross	97.81

**Table 13:** Contribution of individual shallow features on AID (50% training).

Feature Removed	OA Drop (%)	Most Affected Class (OA Drop)
Spatial Coordinates	0.6	Parking Lot (−1.8%)
YCbCr	0.9	CrossCommercial (−2.1%)
GLCM	1.5	Forest (−3.7%)
Canny	1.8	Bridge (−4.2%)

**Table 14:** Sensitivity of CBCAM to temperature parameter ττ on the AID (50% training ratio).

Temperature	0.1	0.5	1.0	2.0	5.0
OA (%)	97.32 ± 0.25	97.76 ± 0.20	97.81 ± 0.23	97.78 ± 0.21	97.28 ± 0.27

## Data Availability

The data associated with this research are available online. The RSICB-256 dataset is available for download at https://github.com/lehaifeng/RSI-CB (accessed on 12 May 2025). The AID is available for download at https://captain-whu.github.io/AID/ (accessed on 12 May 2025). The NWPU-RESISC45 dataset is available for download at https://gcheng-nwpu.github.io/#Datasets (accessed on 12 May 2025).

## Abbreviations

The following abbreviations are used in this manuscript:

AID	Aerial Image Dataset
ASM	Angular Second Moment
BCQA	Bi-direction Cross-Query Attention
BoVW	Bag-of-Visual-Words
BN	Batch Normalization
CBAM	Convolutional Block Attention Module
CBCAM	Cosine Similarity-Based Bidirectional Cross-Query Attention Mechanism
CH	Color Histogram
CM	Confusion Matrix
CMT	Convolutional neural networks meet Vision Transformers
CNN	Convolutional Neural Network
Con	Contrast
Conv	Convolution
COR	Correlation
EAM	Enhanced Attention Module
ENT	Entropy
F1	F1 score
FC	Fully Connected
GAL	Global–Local Attention Mechanism
GAP	Global Average Pooling
GLCM	Gray-Level Co-occurrence Matrix
GMACs	Giga Multiply Accumulation operations per Second
HRRSI	High-resolution Remote Sensing Images
HOG	Histogram of Oriented Gradients
IDM	Inverse Difference Moment
KC	Kappa Coefficient
LBP	Local Binary Pattern
LCAB	Lightweight Context-Aware Attention Block
LGDL	Lie Group Deep Learning
LGM	Lie Group Model
LGRIN	Lie Group Regional Influence Network
LiG	Lie Group
LLC	Locality-Constrained Linear Coding
LS-LieNet	Landslide Interpretation Network with Lie Group Features
LWGANet	Lightweight Group Attention Network
MobileNet	Mobile Network
MSFMA	Multi-Scale Feature Fusion and Mixed Attention Mechanisms
MSRes-SplitNet	Multi-Scale Residual Split Network
NWPU-RESISC	Northwestern Polytechnical University Remote Sensing Image Scene Classification
OA	Overall Accuracy
Params	Parameters
PDConv	Parallel Dilated Convolution
ReLU	Rectified Linear Units
ResNet	Residual Network
RSSC	Remote Sensing Scene Classification
RSICB	Remote Sensing Image Classification Benchmark
SCViT	Sparse Coding Vision Transformer
SE	Squeeze and Excite
SENet	Squeeze-and-Excite Network
SeLU	Scaled Exponential Linear Unit
SGD	Stochastic Gradient Descent
SIFT	Scale-Invariant Feature Transform
SPM	Spatial Pyramid Matching
STConvNeXt	SpatioTemporal ConvNeXt
SwinHCST	Swin-Inspired Hierarchical Cross-attention Spatial Transformer with CNN preprocessing
Swin-Transformer	Shifted Window Transformer
UCMerced	UC Merced Land Use Dataset
ViT	Vision Transformer
ViT-B	Vision Transformer-Base Model
ViTAE	Vision Transformer with Advanced Exploration
VLAD	Vectors of Locally Aggregated Descriptor
